# Intracellular Dynamics of Extracellular Vesicles by Segmented Trajectory Analysis

**DOI:** 10.1021/acs.analchem.2c02928

**Published:** 2022-12-13

**Authors:** Kaisa Rautaniemi, Thomas John, Maximilian Richter, Benedikt C. Huck, Jacopo Zini, Brigitta Loretz, Claus-Michael Lehr, Elina Vuorimaa-Laukkanen, Ekaterina Lisitsyna, Timo Laaksonen

**Affiliations:** † Chemistry and Advanced Materials, Faculty of Engineering and Natural Sciences, Tampere University, Korkeakoulunkatu 8, 33720Tampere, Finland; ‡ Experimental Physics, Saarland University, 66123Saarbrücken, Germany; § Helmholtz Institute for Pharmaceutical Research Saarland (HIPS), Saarland University, Campus E8 1, 66123Saarbrücken, Germany; ∥ Department of Pharmacy, Saarland University, 66123Saarbrücken, Germany; ⊥ Drug Research Program, Division of Pharmaceutical Biosciences, Faculty of Pharmacy, University of Helsinki, Viikinkaari 5, 00790Helsinki, Finland

## Abstract

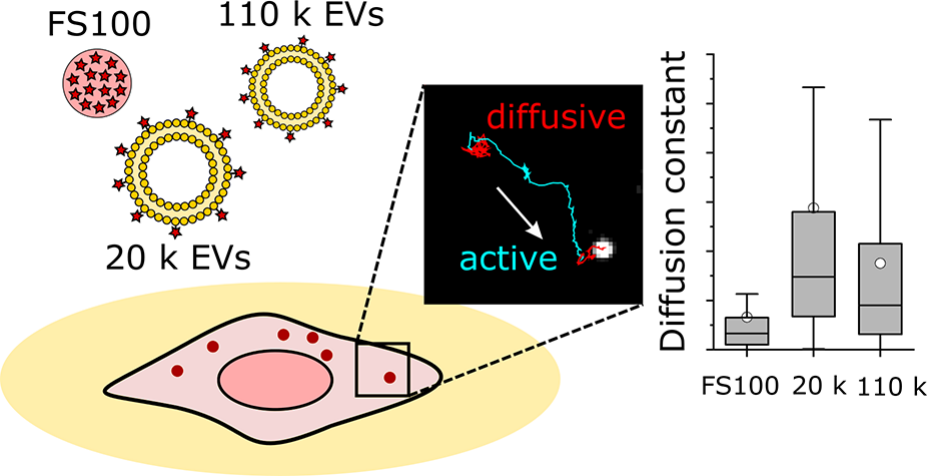

The analysis of nanoparticle (NP) dynamics in live cell studies by video tracking provides detailed information on their interactions and trafficking in the cells. Although the video analysis is not yet routinely used in NP studies, the equipment suitable for the experiments is already available in most laboratories. Here, we compare trajectory patterns, diffusion coefficients, and particle velocities of NPs in A549 cells with a rather simple experimental setup consisting of a fluorescence microscope and openly available trajectory analysis software. The studied NPs include commercial fluorescent polymeric particles and two subpopulations of PC-3 cell-derived extracellular vesicles (EVs). As bioderived natural nanoparticles, the fluorescence intensities of the EVs limited the recording speed. Therefore, we studied the effect of the recording frame rate and analysis parameters to the trajectory results with bright fluorescent commercial NPs. We show that the trajectory classification and the apparent particle velocities are affected by the recording frame rate, while the diffusion constants stay comparable. The NP trajectory patterns were similar for all NP types and resembled intracellular vesicular transport. Interestingly, the EV movements were faster than the commercial NPs, which contrasts with their physical sizes and may indicate a greater role of the motor proteins in their intracellular transports.

## Introduction

Tracking the interactions of nanoparticles (NPs) with cells and tissues is a vital part of understanding their functionalities. It is usually studied by imaging methods, especially by confocal microscopy, providing a general map of the NP distribution inside cells. However, by using microscopy methods with fast image acquisition, it is possible to create videos of the particle movements and therefore follow the fate of a single NP in high temporal resolution, enabling detailed analysis of the NP dynamics.^1^ In biological fields, the trajectories are frequently interpreted mainly visually,^2−4^ which may offer a high amount of information but is laborious and sensitive to the scientist performing the analysis. In contrast, quantitative analysis of the particle trajectories provides information on the diffusion coefficients, velocities, and directions of the particle movements in the cells, which is valuable for the interpretation of the fates of nanocarriers inside cells and how they interact with intracellular components.^5−7^

Several different transport modes have been recognized for the NP trajectories in the cells. In addition to free Brownian motion (normal diffusion) driven by thermal fluctuations, NPs undergo active transport, which originates from the transport driven by motor proteins along the cytoskeletal network.^8−10^ In crowded environments, such as membrane structures and cellular fluids containing obstacles and traps hindering the NP movements, the NP transport may be characterized by anomalous subdiffusion.^11−13^ In this case, the apparent movements of NPs are slower than with free Brownian motion. In video tracking, this mode may also result from experimental suboptimality, such as the localization error.^11,14^ Usually, each NP undergoes different transport modes in a time scale of seconds or even subseconds,^15,16^ resulting in complex dynamical analysis of single-particle trajectories. For example, the intracellular trafficking of exosomes and the uptake of peptide-coated NPs have been described by “stop and go” movement,^2,17^ and the vesicular movement within the cell has been reported to contain alternating diffusive and active steps.^15,16^ Therefore, a simple analysis of the mean squared displacements (MSD) over the whole trajectory, which can be used, for example, for studying the rheological properties of biological fluids,^18^ does not reveal the complete truth of the particle dynamics in the cells.^19^ Generally, these kinds of trajectories should be analyzed by identifying segments of different transport modes from single trajectories.^15,16,20^

An increasing number of advanced imaging methods, including near-infrared surface-enhanced Raman scattering imaging,^21^ 3D holographic fluorescence imaging,^22^ and two-photon laser scanning microscopy,^17^ have been demonstrated to be attractive for the NP tracking studies. However, the particle dynamics can be studied by virtually any microscopic setup offering high enough spatiotemporal resolution. Here, we demonstrate the possibilities of the video tracking method with a rather simple experimental setup: we recorded the trafficking of NPs in A549 cells with a widefield fluorescence microscope and used an openly available particle detection and linking tool by Tinevez et al.^23^ together with a trajectory segmenting tool by Wagner et al.^20^ for studying their interactions. Three types of NPs were used: commercial bright fluorescent polystyrene particles and two subpopulations of PC-3 cell-derived extracellular vesicles (EVs) differing in size. The particles represent very different NP types: polystyrene particles are engineered polymeric particles and therefore have highly controlled properties, high fluorescence intensity, and high uniformity and served the purpose of a control sample. In contrast, EVs are a heterogeneous group of biological nanoparticles secreted by cells. They are an attractive alternative for drug delivery as they are well tolerated in the circulation and seem to have innate targeting properties.^24^ However, the mechanisms and dynamics of their cellular uptake and trafficking are not yet fully understood, making them an interesting subject for the video tracking studies.

## Materials and Methods

### EV Isolation and Characterization

PC-3 cell (ATCC, USA)-derived extracellular vesicles were produced in a bioreactor and isolated with differential ultracentrifugation followed by further purification with discontinuous density gradient. The EV isolation and following characterization are summarized in S1 and described in detail in ref (25). In this study, the EV populations are classified as 20 k EVs and 110 k EVs, according to the forces in units of *g* used for their isolation. The EV subpopulations differed in size, as 20 k EVs were larger (mean diameter 210 nm) than 110 k EVs (mean 150 nm) when measured with nanoparticle tracking analysis (NTA).^25^

### EV Labeling for Tracking Experiments

The EVs were labeled with Alexa Fluor 594 NHS ester (Jena Bioscience, Germany; from now on AF594). The NHS ester group of the AF594 dye forms a covalent bond with the amine groups present on the proteins on the EV surface.^26,27^ About 10^11^ EVs were incubated for 1 h in RT with AF594 (61 μM labeling concentration) in Dulbecco’s phosphate buffered saline (DPBS). As a control, additional EV samples were labeled similarly with inactivated AF594 to ensure that the dye is covalently attaching to the EV membrane. The removal of the unbound dye from the EVs was done by size-exclusion chromatography. The labeling protocol, control studies, and size-exclusion chromatography are described in S1. The particle concentrations and the size distributions of the isolated EV fractions were analyzed using a NanoSight LM-14 instrument (532 nm laser, Nanosight, United Kingdom), equipped with a EMCCD camera (Andor Luca DL 568m-OEM). The samples were diluted with DPBS and measured using a camera level 15 and an acquisition time of 30 s. Three videos were recorded for each sample. The resulting videos were analyzed using the NanoSight NTA software (NanoSight Ltd., v. 3.3) with a detection threshold set to 5. The particle concentrations were used for calculating the EV recoveries. The dye recoveries and labeling efficiencies were measured by a plate reader (Tecan Infinite 200 Pro, Tecan Trading AG, Switzerland) against a calibration curve of known AF594 concentrations in DPBS.

### Video Tracking

A549 (ACC 107, DSMZ, Germany) lung cancer cells were used for video tracking. Cells were cultivated in RPMI 1640 medium with l-glutamine and 10% fetal bovine serum (FBS). The medium was changed every 2 to 3 days and weekly passaging at confluency of approximately 80%. For the experiments, 10,000 or 20,000 A549 cells were seeded on each well of an 8-well chamber plate (n:o 1.5 coverslip bottom; Ibidi, Germany) 3 or 2 days before the imaging, respectively, in phenol red free RPMI media supplemented with 10% FBS. On the imaging day, the medium was changed, and 100 μL of EV suspension (1 × 10^9^ to 2 × 10^9^ EVs) or 10^9^ FluoSpheres (carboxylate-modified, diameter 100 nm, excitation/emission maxima 580 nm/605 nm; ThermoFisher Scientific; from now on FS100) were applied to each studied well. The samples were incubated with the particles for 3 h prior to imaging. The EV samples were washed once with prewarmed media to remove particles not attached or taken up by cells and imaged in fresh media. FS100 samples were imaged without the media change, and the particles not incorporated into the cells were separated in the analysis step.

Microscopy was done at room temperature with an inverted fluorescence microscope (Nikon Eclipse Ti–S) equipped with a Nikon Intensilight 130 W mercury lamp (excitation 562/40 nm and emission 624/40 nm) and a 60× oil immersion objective (NA = 1.4). The tracking videos were recorded with an Orca R2 monochrome 1.3 MP CCD camera (Hamamatsu) at a resolution of 0.092 μm per pixel. FS100 in the A549 cells was recorded with three different frame rates (50, 20, and 2 fps). Cells with a high number of fluorescence spots were chosen for the video recording to have enough trajectories for the analysis. Due to the low initial brightness and photobleaching during the recording, the recording rate and length of the EV videos were limited to 2 fps.

### Theory and Data Analysis

The 2D projections of the particle positions (*x*_j_,*y*_j_) were detected from the image sequences and combined into trajectories. Each trajectory *i* consists of *N*_*i*_ frames, and the time interval between the frames is Δ*t* = (fps)^−1^. A time lag τ is defined as *τ = k*Δ*t*, where *k* = 1, ..., *N*_*i*_ – 1. The squares of the spatial displacements
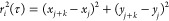
1from trajectories *i* were used to calculate the *time-averaged* mean squared displacement over the whole trajectory with each τ^28^
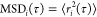
2

For larger τ, the accuracy in MSD_*i*_(*τ*) decreases, as well as for too short of trajectories.^29^ Therefore, only the sufficiently long trajectories were considered in the analysis (Table 1). The average over *n* single MSD_*i*_ defines the *ensemble-averaged* mean squared displacement
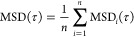
3

**Table 1:** Video Lengths, Analysis Parameters, and Number of Analyzed Trajectories for Videos of FS100 and EVs in A549 Cells^a^

	FS100 in cells						110 k EVs in cells	20 k EVs in cells
Frame rate (fps)	50	50	20	20	2	2	2	2
Video length (s)	20	20	30	30	250	250	250	250
Min. traj. length (s)	1	5	5	15	15	25	25	25
Analysis window (s)	1	5	5	15	15	25	25	25
Min. segment (s)	0.6	3	3	9	9	15	15	15
Tracks analyzed	52	48	52	47	70	61	130	107

a FS100 videos in cells were recorded with frame rates 50, 20, and 2 fps and analyzed with two analysis window sizes. EV tracks were recorded only with 2 fps due to their low initial brightness. Three videos were analyzed for each frame rate.

Typically, the classification of the trajectory movement mode is based on the shape of the extracted MSD_*i*_, defined asnormal diffusion:
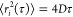
4active motion with diffusion:
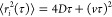
5anomalous subdiffusion:
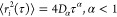
6confined diffusion:
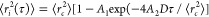
7In the equations above, *D* is the diffusion coefficient, *v* the velocity of the active movement of the particle, and α the anomality coefficient. For the confined motion, *r*_*c*_ is the confinement radius, and constants *A*_1_ and *A*_2_ define the shape of the confinement.^12^Equation 6 can also be used to classify the transport mode: for normal diffusion, α = 1, for superdiffusion, which is often related to the active motion, α > 1, and for subdiffusion, α < 1.^20^ In other words, MSD(τ) for larger time lags is larger for superdiffusion and smaller for subdiffusion than expected for the normal diffusion. Another characteristic property for diffusion is the probability density function (PDF) of the displacements after a certain time lag τ. For normal diffusion, a Gaussian behavior with increasing standard deviation is expected, while for the other movement modes, the PDF deviates strongly from a parabular in the semilogarithmic plot.

We used two approaches for the analysis. In the first part, the complete trajectories were analyzed without segmenting. The relative particle displacements were calculated for each trajectory as Δ*x* = max (*x*_*i*_) – min (*x*_*i*_) (Δ*y* similarly) and scaled with the square root of the trajectory duration. This was used to classify the particles into two groups, here referred to as fast and slow particles. Furthermore, the MSDs were fitted with eq 6 to study the distributions of the anomality coefficient and diffusion coefficient, and the Gaussianities of PDFs of the particle displacements were studied. The nonsegmented trajectory analysis was done with homemade Matlab scripts.^30^

**Scheme 1:**
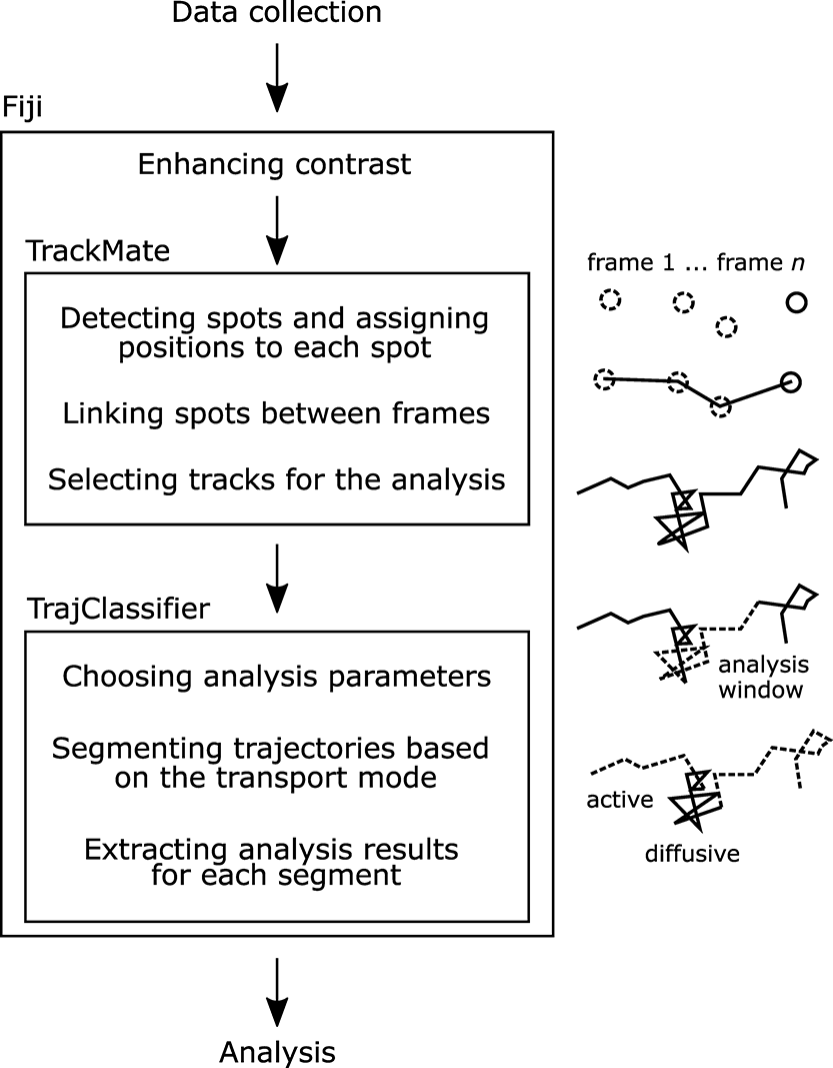
Workflow of Video Analysis

A more advanced analysis of the trajectories including segmenting by the transport modes was performed using the open-source software ImageJ2/Fiji with the plugins TrackMate^23^ and TrajClassifier.^20^ The overall workflow is presented in Scheme 1. The trajectories were first constructed with TrackMate and then segmented with TrajClassifier. Before detecting the particles in an image sequence, the brightness and contrast were adjusted in Fiji (FS100: brightness and contrast tool, EVs: enhance contrast). The spots were identified with LoG detector with an estimated blob diameter 0.7 μm (FS100) or 1.0 μm (EVs) and allowing subpixel localization. The linking of the spots was done by the simple LAP tracker (linking and gap-closing max distance 0.5–1.0 μm and gap-closing max frame gap 2). Then, clear false trajectories, caused by, e.g., diffraction rings, were manually removed, and the trajectory fragments were manually combined to form single complete trajectories when possible. The manual editing of the trajectories was especially necessary for the EV videos, as the EVs were initially dimmer than FS100, leading to lower quality videos. The resulting trajectories were exported to TrajClassifier for segmenting and further analysis.

TrajClassifier uses the rolling window approach for the analysis; i.e., only the part of the trajectory inside the analysis window is analyzed at a time. One of the four trafficking modes (diffusive, active, subdiffusive, or confined) is assigned to each position of the trajectory, and after the whole trajectory is analyzed, the positions with the same mode are combined to the segments.^20^ To study the effect of the number of detected spots within the same time window, the same analysis window size (in seconds) was used for two different frame rates. The minimum segment lengths were chosen to be 60% of the analysis window size. The lengths of the recorded videos, analysis window sizes, minimum segment and trajectory lengths, and numbers of analyzed trajectories are presented in Table 1. For all analyzed videos, the possible drift was subtracted with TrajClassifier. The total trajectory lengths, classified segment lengths, diffusion constants, and active segment velocities were extracted from the analysis software, and the relative amounts of positions classified to each transport mode were calculated from the segment and trajectory lengths.

## Results and Discussion

In an ideal video tracking experiment, several temporal and spatial scales are covered within a single trajectory enabling the reliable determination of the transport mode and the related dynamical parameters. This is achieved when the recording rates are fast (tens of frames per second) and continuous trajectories in a single transport mode long (minimum of hundreds of positions; several minutes in time). Furthermore, the particles should move long enough distances that the localization error does not affect the MSD analysis.^28,29^ However, the intracellular distances are small, and many of the intracellular events last only short moments, making it difficult to capture enough positions in one transport mode and in high enough resolution for very deep analysis. Consequently, this type of analysis requires at least some level of compromise. In this study, we were limited to a 2 fps recording rate with the EVs due to their low fluorescence intensities and therefore expected to lose some information of the dynamics taking place in subsecond to few second time scales. The recording rate directly affects the time scales available for the analysis and, consequently, the resulting MSDs. To understand how changing the frame rate affects the classification and parameters extracted from the trajectories, we studied bright fluorescent and photostable commercial nanoparticles in the A549 cells with three different recording rates before tracking the EVs.

### Nonsegmented Trajectory Analysis

**Figure 1:**
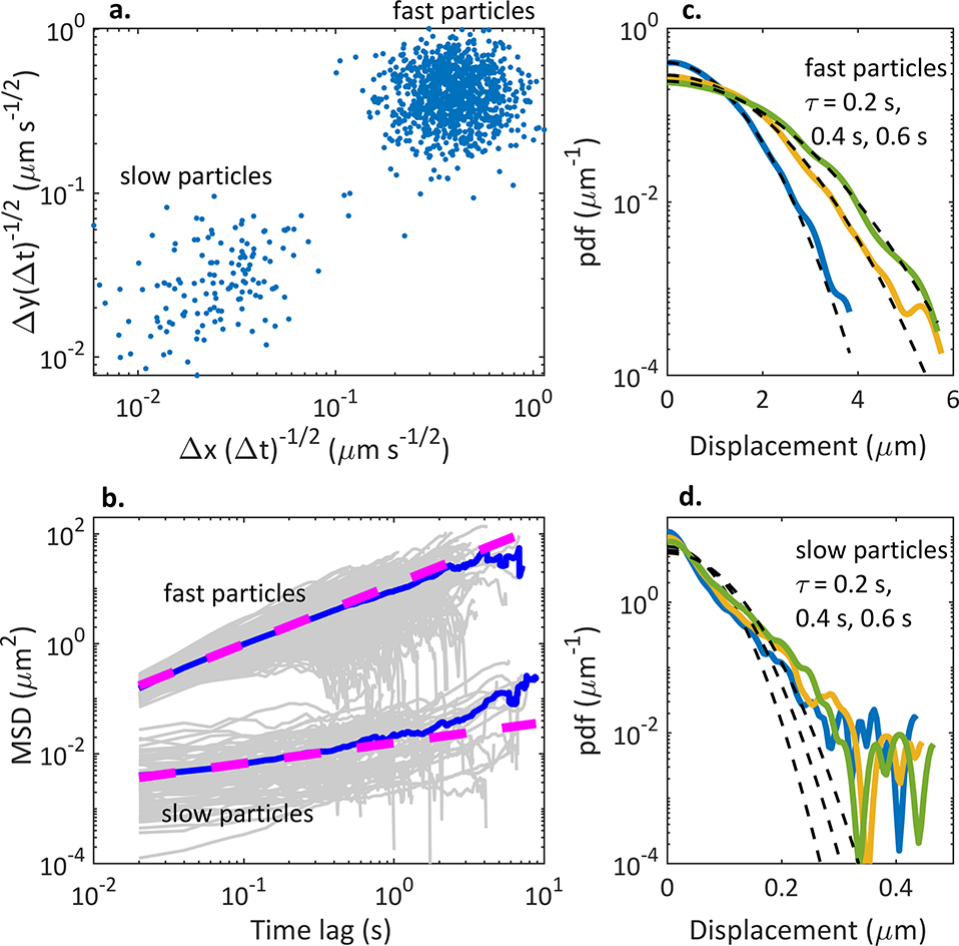
Nonsegmented trajectory analysis of the cell sample recorded with 50 fps. (a) Relative displacement of the trajectories show two particle groups: fast particles outside the cells (upper right corner) and slow particles within the cells (lower left corner). (b) MSDs of fast and slow particles. Ensemble average is shown in blue and fit to the average in magenta. (c, d) Probability density functions for the FS100 displacements for fast and slow particles. The experimental result is shown with solid lines and the Gaussian fit in dashed lines.

In a representative 50 fps cell sample video, the particle trajectories formed two distinct groups, fast and slow particles, by relative displacement in a square root of time (Figure 1a) and in MSD curves (Figure 1b). The fast particles were distributed over the whole imaged area in the cell culture media, while the slow particles co-located with the cells. For each trajectory, the experimental MSD_*i*_ (Figure 1b, gray lines) was calculated and fitted to eq 6. The fast particles showed a Brownian behavior, seen as α_*i*_ narrowly distributed around 1 (Figure S1a) and the Gaussian shape of the PDF of the displacements (Figure 1c). The diffusion constant obtained from the ensemble-average MSD was 3 μm^2^ s^–1^ (Figure S1d), giving a viscosity of 1.4 mPa·s (by Stokes–Einstein relation, *D = k*_B_*T*(3πη*d*)^-1^) which is close to water viscosity of 1 mPa·s at room temperature. As the assumed particle environment was homogeneous (cell culture media), the obtained diffusion and anomality coefficients from the MSD_*i*_ were expected to be distributed due to the finite length of the experimental trajectories.^29^ To prove this, we simulated trajectories with Brownian motion, using the obtained mean diffusion coefficient but the same number and individual lengths as in the experimental data set (Figure S2). The diffusion *D*_*i*_ and anomality coefficients α_*i*_ from the simulated trajectories show almost the same distributions as from the experimental data (Figure S1a and d), confirming the homogeneity of the particle environment.

The slow particles showed clearly more complex dynamics than the fast particles. The distribution of α_*i*_ (Figure S1b) was broad with maxima at 0.4 and 1.4, indicating the presence of subdiffusive and superdiffusive movement modes, respectively. Furthermore, the shape of the PDF did not follow the Gaussian dynamics (Figure 1d), suggesting that more detailed analysis was required. A similar analysis was repeated for a 2 fps video (Figures S3, S1c and f). Only the slow cell-associated particles could be tracked with this low frame rate as is seen in the relative displacements (Figure S3a). Both the distribution of α_*i*_ (Figure S1c) and the PDF of displacements (Figure S3c) resembled the slow particles recorded with 50 fps, indicating the presence of different movement mode trajectories. The mean diffusion constant values were only 0.004 μm s^–2^ (50 fps) and 0.0008 μm s^–2^ (2 fps) with broad distributions (Figure S1e and f). These would give cellular viscosities in the range of Pa·s, thousand times as large as in water. The viscosity range is similar as has been reported previously for different cell types.^31^

### Segmented Trajectory Analysis

**Figure 2:**
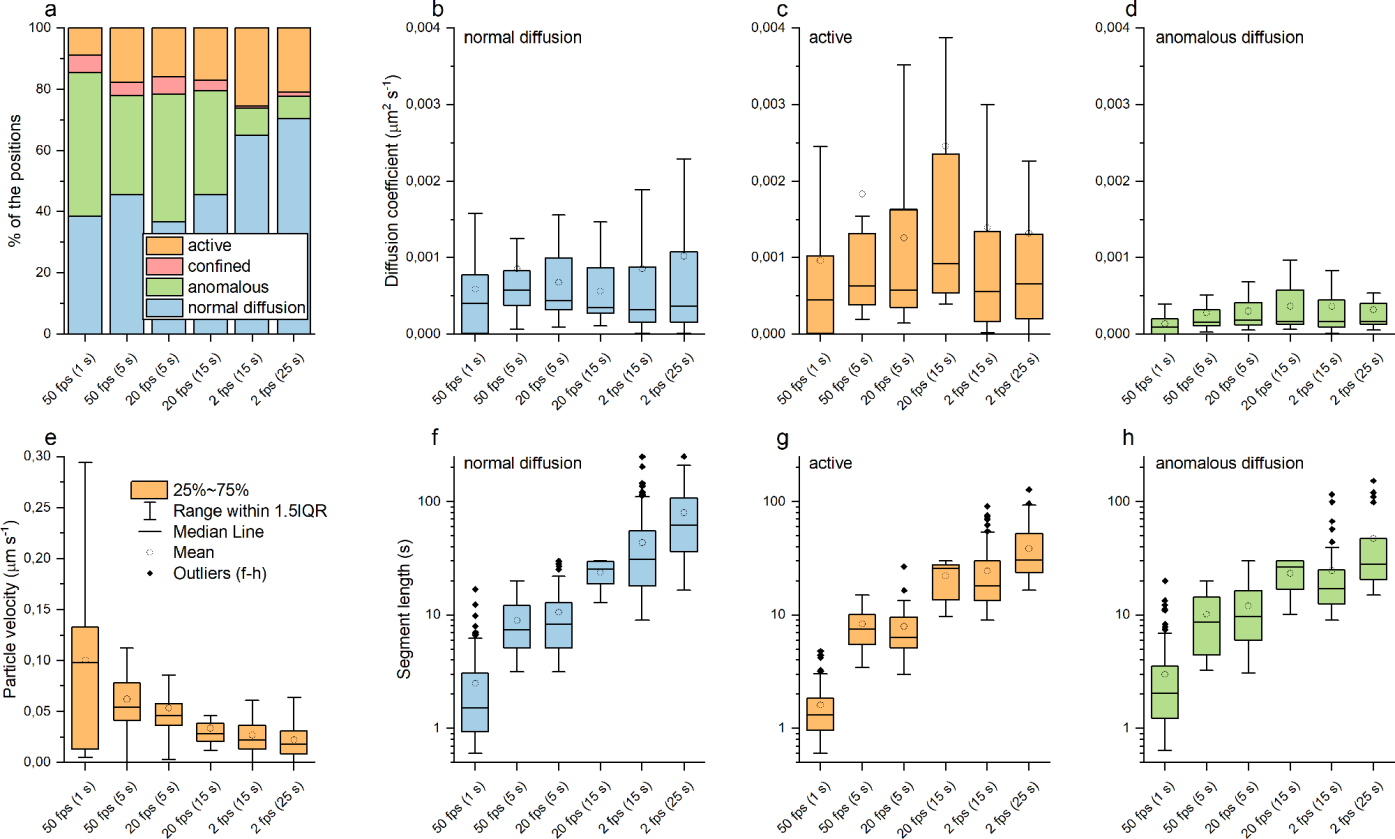
FS100 dynamics in A549 cells recorded with 50, 20, and 2 fps rates and analyzed with two window sizes each (in brackets). (a) Distributions of positions categorized to different transport modes, (b–d) diffusion constants, (e) particle velocities for active segments, and (f–h) segment lengths. For scaling reasons, outliers are not presented in b–e.

For a more detailed analysis, the trajectories within the A549 cells were segmented based on the transport modes. Since the intracellular trajectories are complex containing segments of different movement modes and directions, and each MSD(τ) averages information over the whole trajectory, segmenting the trajectory with a proper analysis window is essential for identifying the underlying dynamics. Here, we used the analysis windows from 1 to 25 s, and the same analysis window size was used for two different frame rates (Table 1). The average α values of the transport modes for each set of parameters are presented in Table S1. The proportions of the positions categorized to different transport modes, velocities for the active segments, diffusion coefficients, and segment lengths are presented in Figure 2. All four movement modes were detected in A549 cells. The proportion of confined motion was small (Figure 2a), and we excluded this mode from further analysis.

The recording frame rate and analysis window show clear effects on subsequent trajectory segmentation and particle velocities (Figure 2a and e). On short time lags, the active movement term in eq 5 is small compared to the diffusive term, and the overall MSD appears to be similar to normal diffusion. Therefore, the analysis window needs to contain long enough time lags to recognize the active segments. The proportions of the active positions are approximately 20% for all the analysis window sizes starting from 5 s (Figure 2a), suggesting that 5 s is a sufficient threshold value in our videos. The classification to active mode in 2 fps videos was more in line with the higher frame rates when a 25 s analysis window was used instead of a 15 s window. Interestingly, the proportions of the positions classified as anomalous diffusion decrease remarkably even with the same 15 s analysis window when the frame rate is decreased from 20 to 2 fps, showing that anomalous diffusion appears as normal diffusion on longer time scales. Based on these results, the optimal settings for detecting both active movement and anomalous diffusion in a same video with high time resolution would be using at least a 20 fps recording rate and 5 s analysis window.

The active segment particle velocities systemically decreased with both the decreasing frame rate and increasing analysis window (Figure 2e), from a median of 0.1 μm s^–1^ (50 fps, 1 s) to 0.02 μm s^–1^ (2 fps, 25 s). Previous studies suggest that the myosin-driven transport along microfilaments occurs at velocities mainly below 0.5 μm s^–1^, while kinesin- and dynein-driven transports along the microtubules can have velocities of a few μm s^–1^.^10,32,33^ However, also slow microtubule-mediated cellular transport has been reported.^8^ Therefore, the observed particle velocities indicate most of the detected particles moving either along the microfilaments or slowly along the microtubules. The diffusion constants (Figure 2b–d) showed a wide distribution in cells, reflecting the presence of several different intracellular microenvironments. Although the diffusion constants had some variation with the experimental parameters, median values were still comparable regardless of how the data were obtained, and no systematic change was observed. The diffusion constants were mainly less than 0.002 μm^2^ s^–1^ (normal diffusion), 0.004 μm^2^ s^–1^ (active), and 0.001 μm^2^ s^–1^ (anomalous diffusion) (Figure 2b–d). Similar to particle velocities, the diffusion constants were close to those reported for the transports along the microfilaments^10,32,33^ or slow transports along the microtubules.^8^ The same order of magnitude values have also been observed for the slow EV movements on the cell surface.^27^

The effect of the analysis window size to the segment lengths is shown in Figure 2f–h: the median of the segment length was similar with the same size of analysis window regardless of the frame rate, and the segment lengths increased with increasing window size as expected. In other words, shorter consecutive segments of different modes were categorized as a single segment of one transport mode with a larger analysis window, which can clearly be seen in Figure S4. This may also partly explain the decrease of the particle velocities for the active segments with increasing analysis window (Figure 2e): segments classified as “active” contain also shorter segments of slower movement modes, decreasing the average particle velocity.

For comparison with the NP trajectories in the cells, we tracked FS100 in a simpler media, 1% hydroxyethyl cellulose with a 50 fps rate, and analyzed a representative video by both analysis approaches (S2). Both the nonsegmented and segmented trajectory analyses showed almost only normally diffusive behavior with identical diffusion coefficients, and the resulting microrheological viscosity value was close to the macroscopic viscosity of a similar sample. Based on these results, the particle dynamics in the cells clearly differ from a nonactive simple viscous solution and can be further segmented and classified to different transport modes. However, the sensitivity of trajectory segmentation, the extracted particle velocities, and segment lengths depended on the experimental and analytical parameters. With 2 fps, the particle velocities in active movement appeared slower, and most of the subdiffusion was not recognized. Nevertheless, the overall diffusion constants did not show dependence on the experimental parameters, and the proportions of the active positions were similar with all frame rates with a large enough analysis window.

Importantly, if the aim of the study is to follow single NPs to understand their intracellular trafficking and fate, the desired trajectory lengths are rather minutes than seconds. To achieve videos lasting several minutes, low frame rates that enable lower excitation power and consequently less photobleaching might be necessary, even at the cost of less precise segment classification.

### Extracellular Vesicle Dynamics in A549 Cells

Finally, we applied fluorescently labeled EVs to the A549 cells to compare their dynamics to the commercial polymeric nanoparticles. Fluorescently labeled EVs are a more challenging tracking target than polymeric NPs as they need to be first reliably labeled with a fluorescent dye.^25^ Because of their bilayer membrane structures, they cannot bind as much of the dye as is present in polymeric NPs, leading to a lower initial brightness. Here, we used bright fluorescent, amine-reactive AF594-NHS ester dye to covalently label the EVs. Based on the characterization of the labeled EVs and the labeling controls (S1), the labeling of EVs was considered successful, and the vesicles were used in video tracking experiments. Regardless of their lower fluorescence intensity compared to FS100, we were able to track the EV movements for tens of EVs per video with 2 fps rates even up to 250 s before they were lost due to photobleaching.

#### Comparison of EV and FS100 Dynamics in A549 Cells

**Figure 3:**
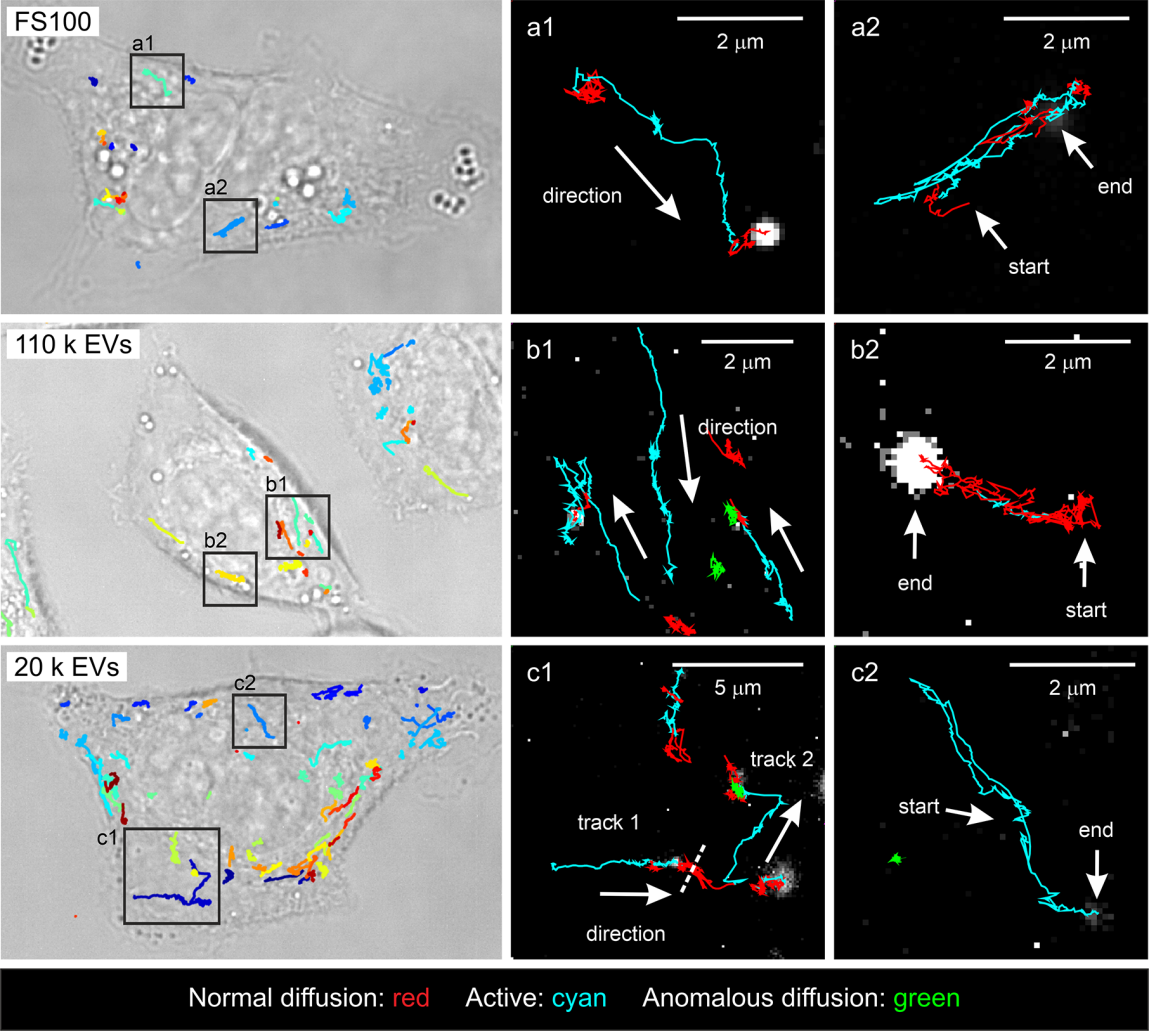
Examples of FS100 and EV trajectories in A549 cells after 3 h incubation. Brightfield microscope images of the cells overlapped with the NP trajectories on the left, and the magnifications of the squares (a1–c2) on the right. The colors in the magnifications represent the transport mode and are explained in the bottom of the figure. No segmentation is shown in the brightfield images.

**Figure 4:**
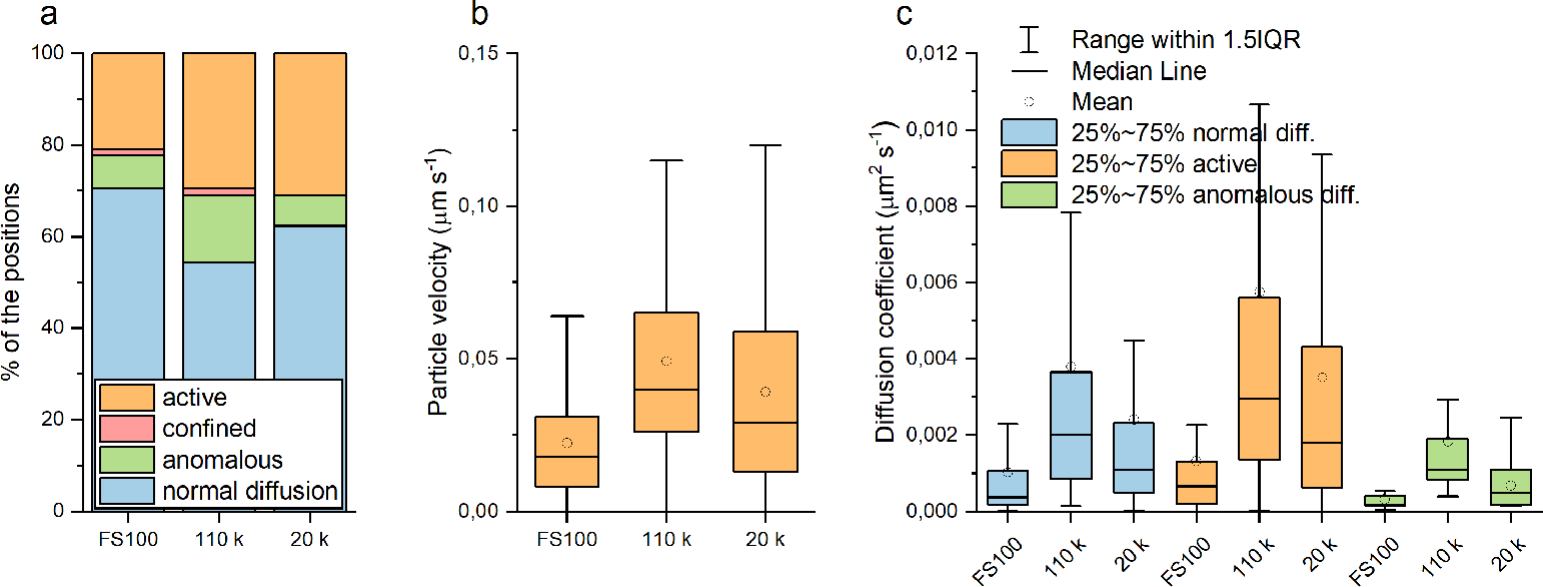
Comparison of FS100, 110 k EV, and 20 k EV dynamics in the A549 cells recorded with 2 fps rates and analyzed with a 25 s window. (a) Distribution of the positions categorized to different transport modes, (b) active segment particle velocities, and (c) diffusion constants. For scaling reasons, outliers are not presented in (b) and (c).

All the EV trajectories were analyzed using a 25 s analysis window and compared with the corresponding FS100 results. Examples of the trajectories in the cells are presented in Figure 3. For the segmented particle trajectories, several NP transport patterns were observed: (1) relatively straight, monodirectional trajectories with alternating active and diffusive or anomalous segments (Figure 3 a1, b1, and c1), (2) back-and-forth moving particles with different proportions of active and diffusive movement in the same trajectory (Figure 3 a2, b2, and c2), and (3) plain diffusion or anomalous diffusion trajectories with almost no observed change in the location (Figure S5). Type 1 movement has been related to intracellular vesicular transport along the cytoskeletal network^16,34^ and trafficking of endocytosed EVs^2,7^ and viruses.^10,32^ The nonactive phases of this movement type may relate to interactions with cellular organelles, such as other endosomes and endoplasmic reticulum^2,34^ or to the intersections in the cytoskeletal network.^32^ Type 2 movement along the same microtubules has been observed previously for the influenza virus.^32^ Type 3 movement has been suggested for the EVs entrapped in lysosomes.^7^ These transport patterns were observed for all the studied NPs, suggesting that the monitored particles were indeed internalized by the cells, and the intracellular trafficking mechanisms are similar for both EV populations and polymeric nanoparticles. The proportions of the positions in different transport modes, active segment velocities, and diffusion coefficients are presented in Figure 4. Regardless of the particle type, the proportions of the different transport modes were similar (Figure 4a). Most of the positions were categorized as normal diffusion, 20%–30% as active movement, and about 10% as anomalous diffusion. Similar to FS100 trajectories, only few EV trajectory segments were classified as confined, and they were excluded from further analysis. The brightness and photostability of FS100 led to long trajectories: most of the trajectories spanned over the whole 250 s video (Figure S6a). However, we were able to record long enough EV trajectories (median values of 61 s/110 k EVs and 103 s/20 k EVs) for the trajectory analysis (Figure S6a). The segment lengths were very similar for all the nanoparticle types (Figure S6b–d). The main difference was that the segments classified as normal diffusion were longer for FS100 than for the EVs. This is probably related to the longer overall trajectories due to more photostable particles. These results support the visual conclusion that the intracellular trafficking is similar for the studied particle types, both EVs and FS100.

Although the movement patterns were similar, differences in particle velocities and diffusion constants between the NP types were observed (Figure 4b and c). The polymeric NPs had both the smallest diffusion coefficients and slowest movements, 20 k EVs had intermediate values, and 110 k EVs expressed the fastest movements. The differences between the EV types may be explained by their different sizes: even after the labeling, 110 k EVs were on average smaller than 20 k EVs (mean diameters 150 nm/110 k and 180 nm/20 k EVs measured by NTA; Figure S8), leading to faster diffusion. The EVs had broader size distributions than FS100, which is reflected in broad distributions of the diffusion coefficients. FS100 had the slowest movements in contrast to their smallest physical sizes, indicating differences in their intracellular trafficking mechanism compared to the EVs. We determined the proportions of active segments possibly in the rapid microtubule-directed transport mode using *D =* 0.01 μm^2^ s^–1^ as the cutoff value.^33^ The cutoff was chosen based on a study comparing microtubule- and microfilament-mediated transport with microfilament-related diffusion values in the same magnitude as our results (10^–3^ μm^2^ s^–1^), although higher cutoff values have also been suggested.^8^ Interestingly, about 14% of 110 k and 11% of 20 k EV active segments could be classified to rapid microtubule-directed modes, while only one segment (<2% of total active segments) for FS100 exceeded the limit. Although the classification is only approximate, the result may indicate a greater role of the motor proteins in the transport along the microtubules for the EVs than for the synthetic polymeric NPs.

## Conclusions

In this study, we demonstrated the analysis of nanoparticle dynamics in live cells with openly distributed analysis software and a readily available microscopic setup. Three NP types were used: commercial highly fluorescent polymeric nanoparticles and two subtypes of PC-3 cell-derived extracellular vesicles. The high fluorescence intensities of the commercial NPs enabled studying the effect of the experimental and analytical parameters to the analysis results. The main findings were as follows: (1) Analysis windows longer than 5 s were sufficient for detecting active movement with all the recording rates. (2) Most of the anomalous diffusion was detected only with the faster imaging rates. (3) The apparent particle velocity decreases with decreasing recording frame rate and increasing analysis window size. (4) The observed diffusion constants are similar for a particular transport mode regardless of the experimental and analytical parameters, making them the most consistent and reliable analyzed parameter. Then, we compared the dynamics of the different NPs in A549 cells both visually and quantitatively. The diffusion constants, particle velocities, and our visual observations of NP movements were similar to what has been described for intracellular vesicle trafficking along the cytoskeletal network, indicating that all the studied NP types were internalized by the cells. Interestingly, the intracellular movements of the EVs were faster than the movements of commercial NPs. However, the results were shown to be sensitive to the experimental and analytical parameters, and therefore, one should be careful when comparing the results from different sources.

The results shown here were achieved by simple instrumentation. Applying a detector with higher sensitivity or generally more modern instrumentation might enable higher time resolutions with similar samples. Furthermore, combining the NP video analysis to organelle labeling would provide even more information of the NP trafficking phases in the endosomal network, enable identifying the organelles that NPs are interacting with, and enable determining the characteristic dynamics for those interactions. This might then reveal the reason for the different observed movement modes. Based on the present research, we highly encourage researchers to study the possibilities of the video analysis and to utilize it more often as a part of NP uptake and trafficking studies.

## Supporting Information Available

Additional result visualizations (Figures S1–S6) and results (Table S1), extracellular vesicle sample preparation (S1), and microrheological and macrorheological characterizations of a control sample (S2) (PDF)
Particle finding and trajectory formation script (MAT) and trajectory analysis script (MAT) (ZIP)
