# Supplementary material for: Intracellular Dynamics of Extracellular Vesicles by Segmented Trajectory Analysis
Source: Anal Chem. 2022 Dec 13;94(51):17770–8. doi: 10.1021/acs.analchem.2c02928 (PMC9798377; doi:10.1021/acs.analchem.2c02928)
Supplement: Supplementary file 1 — ac2c02928_si_001.pdf [file ac2c02928_si_001.pdf]

Supporting information

Intracellular dynamics of extracellular vesicles by segmented trajectory analysis

Kaisa Rautaniemi[a], Thomas John[b], Maximilian Richter[c,d], Benedikt C. Huck[c,d], Jacopo Zini[e], Brigitta Loretz[c], Claus-Michael Lehr[c,d], Elina Vuorimaa-Laukkanen[a], Ekaterina Lisitsyna[a], Timo Laaksonen[e,a]

[a]Chemistry and Advanced Materials, Faculty of Engineering and Natural Sciences, Tampere University, Korkeakoulunkatu 8, 33720 Tampere, Finland; [b] Experimental Physics, Saarland University, 66123 Saarbrücken, Germany; [c]Helmholtz Institute for Pharmaceutical Research Saarland (HIPS), Saarland University, Campus E8 1, 66123 Saarbrücken, Germany; [d]Department of Pharmacy, Saarland University, 66123 Saarbrücken, Germany; [e]Drug Research Program, Division of Pharmaceutical Biosciences, Faculty of Pharmacy, University of Helsinki, Viikinkaari 5, 00790 Helsinki, Finland

Figures S1–S6 .... S-2
Table S1 .... S-5
S1. EV isolation, characterization and AF594 labelling S-5
S2. Polymeric nanoparticle dynamics in viscous solution S-7
References S-10

Figures S1–S6

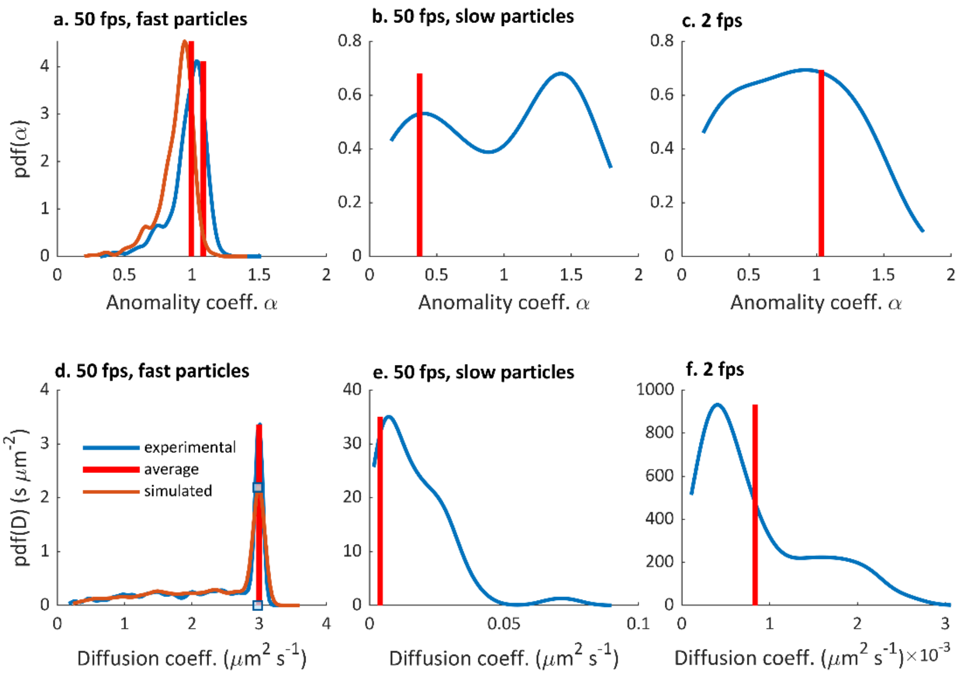

Figure S1. The distribution of anomality coefficients (a–c) and diffusion coefficients (d–f) of FS100 in the A549 cell samples from the non-segmented trajectory analysis. The experimental distribution is shown in blue and the ensemble average as the red vertical line. For the fast particles (50 fps) outside the cells expressing Brownian diffusion, the analysis was repeated with the simulated trajectories (distributions shown in red in a and d): the same number of trajectories with similar lengths as in the experimental data was simulated with Brownian movement and analyzed similarly as the experimental data.

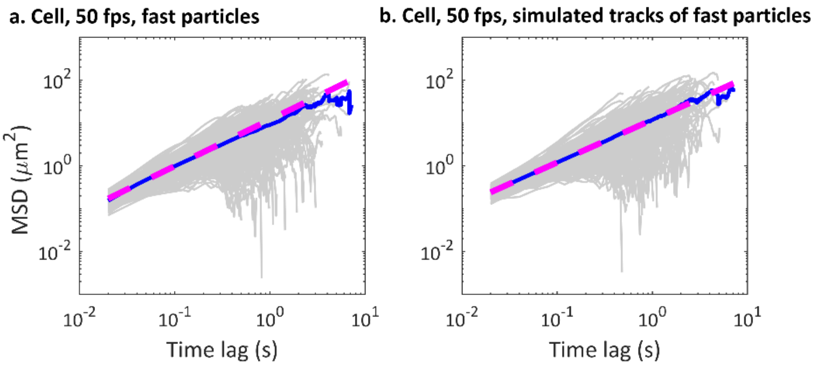

Figure S2. The mean squared displacements (MSD) of the Brownian diffusion in the cell culture media from the non-segmented trajectory analysis (a), and the MSDs derived from the corresponding simulated trajectories (b). Ensemble average is shown in blue and fit to the average in magenta. The same number of trajectories with similar lengths as in the experimental data was simulated with Brownian movement and analyzed similarly as the experimental data. Both experimental and simulated trajectories yielded very similar MSDs, indicating that the experimental trajectories show Brownian movement.

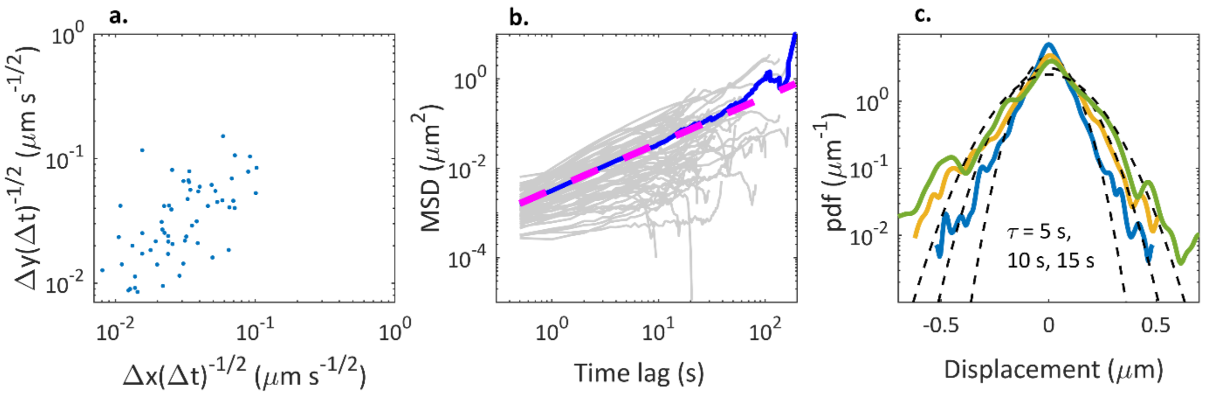

Figure S3. The non-segmented trajectory analysis of the FS100 in A549 cells recorded with 2 fps rate. The relative displacements (a), mean squared displacements (b), and probability density functions with three time lags τ (c). In (b), ensemble average is shown in blue and fit to the average in magenta. With this framerate, only the cell-associated slow particles (lower left corner in a) could be tracked.

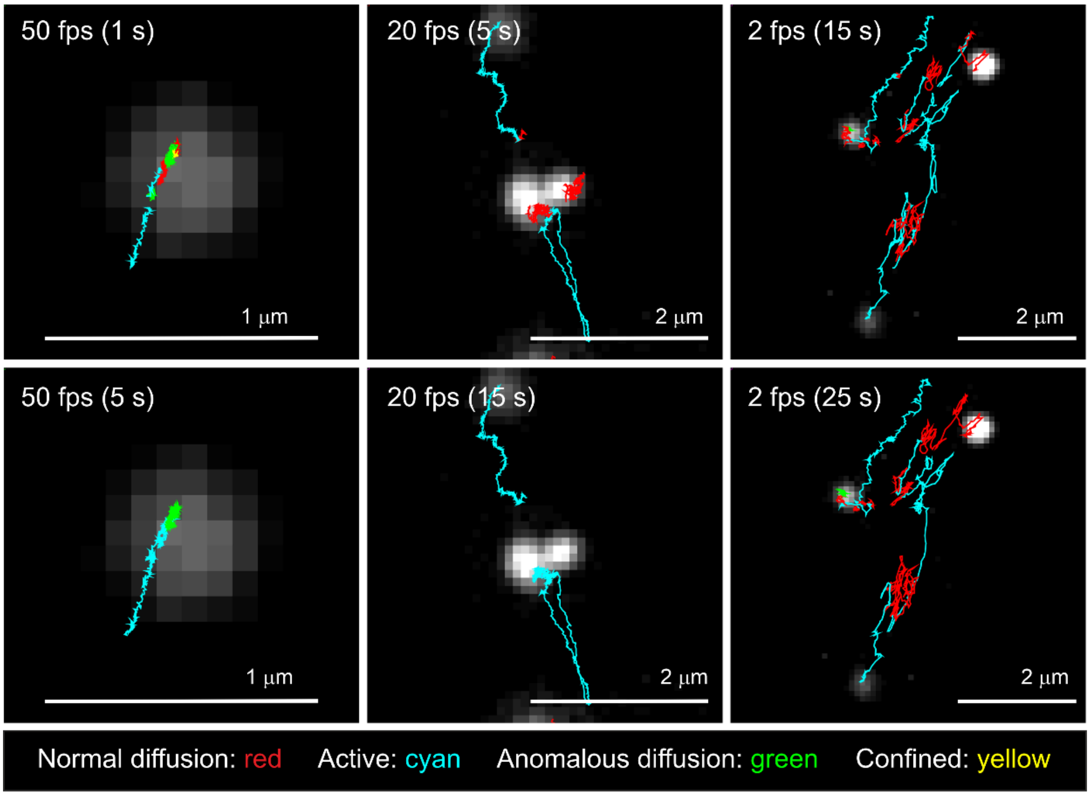

Figure S4. Illustration of how the analysis window size (in brackets) affects the segmentation of FS100 trajectories in A549 cells recorded with 50 fps, 20 fps and 2 fps. With larger analysis window, the shorter segments are not recognized.

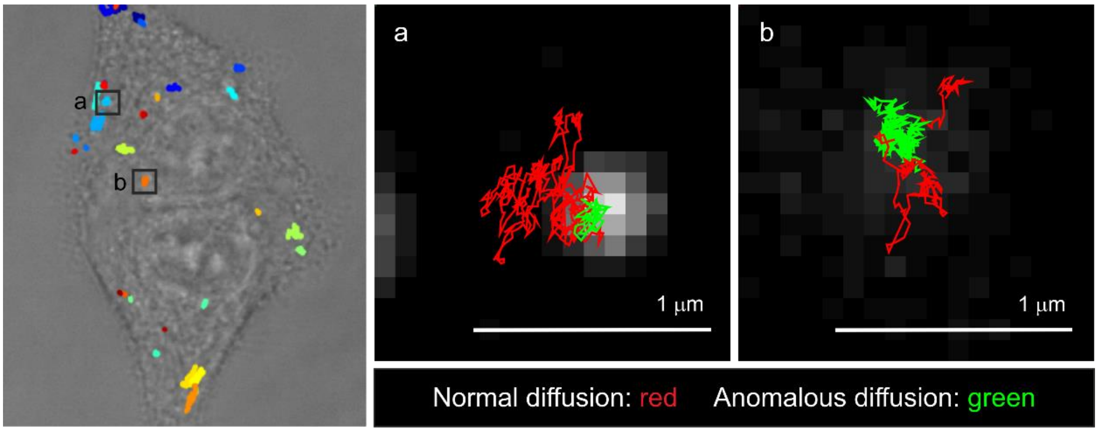

Figure S5. Example of FS100 trajectory consisting of mainly normal diffusion (a) and anomalous diffusion (b) in a A549 cell with almost no observed change in the particle location (type 3 NP transport pattern). The examples were captured with 2 fps rate and analyzed with 25 s window.

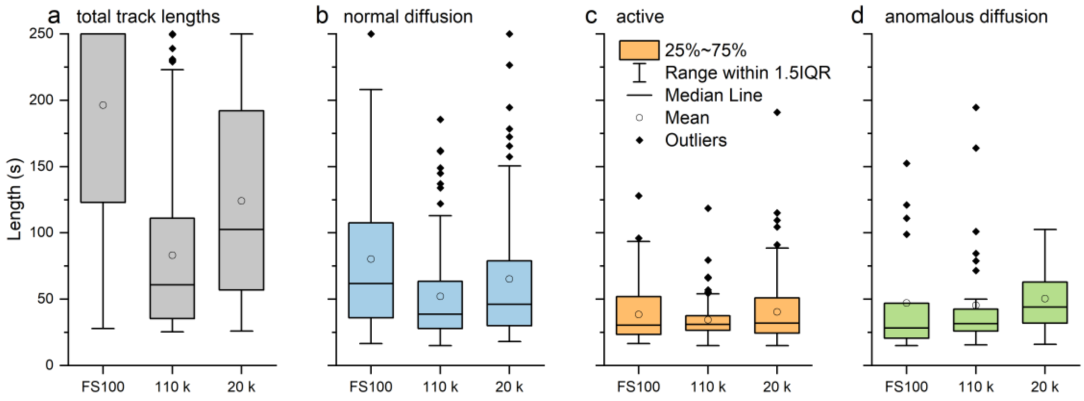

Figure S6. EV trajectory (a) and segment (b–d) lengths in the A549 cells recorded with 2 fps and analyzed with 25 s window. For comparison, the corresponding FS100 results are also presented.

Table S1. The anomality coefficients α for the classified trajectory segments. The values for the subdiffusion and active movement are well in line with the physical description of these transport modes (α > for active and α < 1 for subdiffusion) while they slightly deviate from the theoretical value for the normal diffusion (α = 1). This is explained by the working principle of the trajectory segmenting tool. In addition to the anomalous exponent α, the TrajClassifier classification is based on several other trajectory features: asymmetry, fractal dimension, gaussianity, kurtosis, mean squared displacement ratio, straightness and trappedness.

**NP type**	**Framerate (analysis window)**	**Anomality coefficient α (from the equation $\langle r_i^2(\tau)\rangle = 4D_\alpha \tau^\alpha$)**		
		**Normal diffusion**	**Active**	**Subdiffusion**
FS100	50 fps (1 s)	0.81 ± 0.17	1.20 ± 0.17	0.45 ± 0.16
	50 fps (5 s)	0.86 ± 0.11	1.24 ± 0.13	0.58 ± 0.11
	20 fps (5 s)	0.92 ± 0.14	1.26 ± 0.20	0.48 ± 0.16
	20 fps (15 s)	0.92 ± 0.15	1.28 ± 0.20	0.50 ± 0.15
	2 fps (15 s)	0.94 ± 0.20	1.36 ± 0.21	0.44 ± 0.21
	2 fps (25 s)	0.91 ± 0.20	1.35 ± 0.21	0.44 ± 0.18
110 k EVs	2 fps (25 s)	0.94 ± 0.24	1.37 ± 0.21	0.40 ± 0.18
20 k EVs	2 fps (25 s)	1.01 ± 0.19	1.40 ± 0.26	0.47 ± 0.16
Combined	2 fps (25 s)	0.95 ± 0.21	1.37 ± 0.23	0.43 ± 0.17

S1. EV isolation, characterization and AF594 labelling

PC-3 cell derived extracellular vesicles were produced in a bioreactor and isolated with differential ultracentrifugation followed by further purification with discontinuous density gradient. The isolation and characterization of the EVs are described in detail in ref. [1]. Briefly, the buoyant cells, cell fragments and apoptotic bodies were first removed with low-speed centrifugation. Then, the first EV fraction was pelleted with centrifugation at +4 °C with 20 000 × g for 1 h, and the second EV fraction was collected from the supernatant with centrifugation at +4 °C with 110 000 × g for 2 h. Here, the EV populations are classified as the 20 k EVs and the 110 k EVs, according to the forces in units of g used for their isolation. The obtained EV pellets were resuspended in DPBS buffer and the EVs were further purified with three-layered (0 % – 35 % – 45 %) discontinuous iodixanol density gradient. After the gradient centrifugation, the EVs were collected from the 0 % – 35 % iodixanol interface, and finally, the iodixanol was removed by serial ultrafiltration. The particle concentrations were measured with NTA, and the EV suspensions were divided into aliquots of about 10^{11} particles and stored in –80 °C. The characterization of the isolated EVs was done by NTA, Western blot, transmission electron microscopy and FTIR. The isolated EVs showed high purity and consistently similar properties over each sample replicate. Both EV types showed a broad size distribution from 100 nm to 400–500 nm. The EVs differed in size when measured with NTA: 20 k EVs were larger (mean 210 nm, mode 150 nm) than 110 k EVs (mean 150 nm, mode 120 nm). The bigger size of 20 k EVs is in line with their faster sedimentation during 20 000 × g centrifugation.

Both EV types were covalently labelled with Alexa Fluor 594 NHS ester dye (Jena Bioscience, Germany) (**Figure S7a**). The dye stock (6.1 mM) was prepared in anhydrous dimethyl sulfoxide (DMSO), aliquoted, and stored in –80 °C. About 10^{11} EVs were diluted to a final volume of 100 µl DPBS and mixed with 1 µl of freshly melted AF594 (61 µM labelling concentration), followed by brief vortexing and 1 h incubation at RT upon shaking (300 rpm) covered from light. As a control, additional EV samples were labelled with inactivated AF594 to ensure that the dye is covalently attaching to the EV membrane instead of nonspecific binding to the EVs. For the inactivation, 2 µl of the dye stock was mixed with 8 µl of milli-Q water, 1 µl of 1 M sodium hydroxide, and incubated for 30 min to facilitate the hydrolysis of the reactive group. Then, 5.5 µl of the inactivated AF594 was used for the EV labelling as described above.

The labelled EV suspension was run through a Sepharose CL-2B (GE Healthcare, USA) column (diameter 1 cm, bed size ~13 ml) using DPBS as an eluent. All the runs were performed at room temperature. The eluted buffer was collected in 1 ml fractions starting directly after sample insertion, and a total of 10 fractions were collected for each SEC run. For the first labelled EV samples (both 20 k and 110 k EVs), the particle concentrations of all fractions were measured by NTA and the fluorescence intensities were measured with plate reader (Tecan Infinite® 200 Pro, Tecan Trading AG, Switzerland) for identifying the EV-containing fractions. For the rest of the separations, the EV fractions were identified by fluorescence measurements and the particle concentrations were measured only for the fractions with the highest fluorescence intensities. The separation between the EVs and unbound AF594 was confirmed by a control study without the EVs, collecting 30 fractions of 1 ml each. The size exclusion chromatography offered a good separation of the labelled EVs from the unbound dye: the unbound dye started eluting in fraction 10 (**Figure S7b**), and eluted mainly in the fractions 11–16, while the EVs eluted in fractions 4–6 (**Figure S7b and c**). **Figure S8** shows representative NTA size distributions for the SEC-purified AF594 EVs and the average EV sizes (**Figure S8**, inset). The size distribution is broader and shifted towards larger sizes for 20 k EVs compared to 110 k EVs (mean 180 nm / 20 k and 150 nm / 110 k

EVs) after the SEC purification. The EV concentrations and labelling efficiencies are presented in **Table S2**. The EV concentrations were similar for both covalently labelled EVs and the EVs incubated with the inactivated dye, while the fluorescence intensities of the inactivated labelling control samples were under the detection limit of the instrument, suggesting that the AF594 mostly binds covalently to the EVs. The labelling of EVs was therefore considered successful and the vesicles were used in the video tracking experiments.

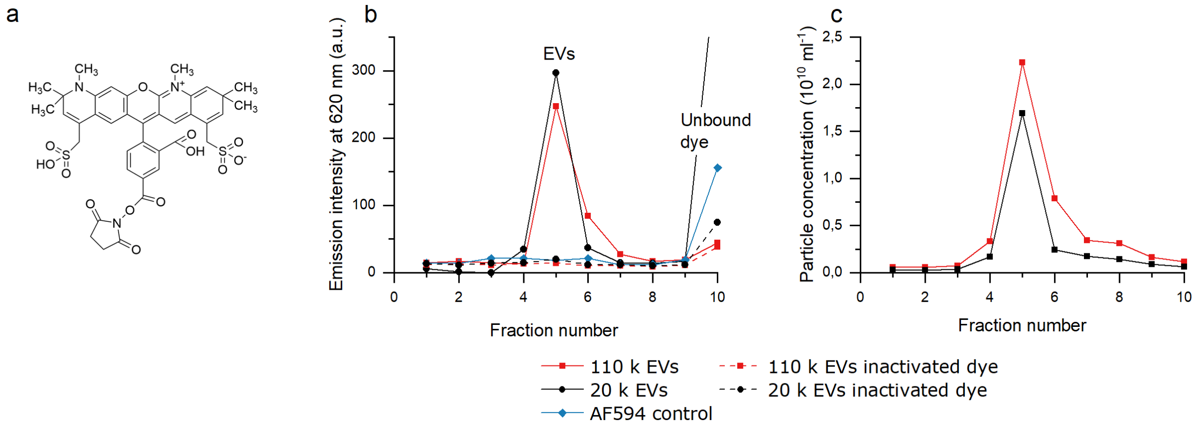

Figure S7. (a) The chemical structure of AF594 NHS ester dye. (b) Fluorescence intensity at 620 nm (550 nm excitation) for the AF594 control without EVs, the EVs labelled with AF594, and the EVs labelled with inactivated AF594. (c) Particle concentration for the AF594 labelled EVs. (b and c) are presented as a function of SEC fraction number.

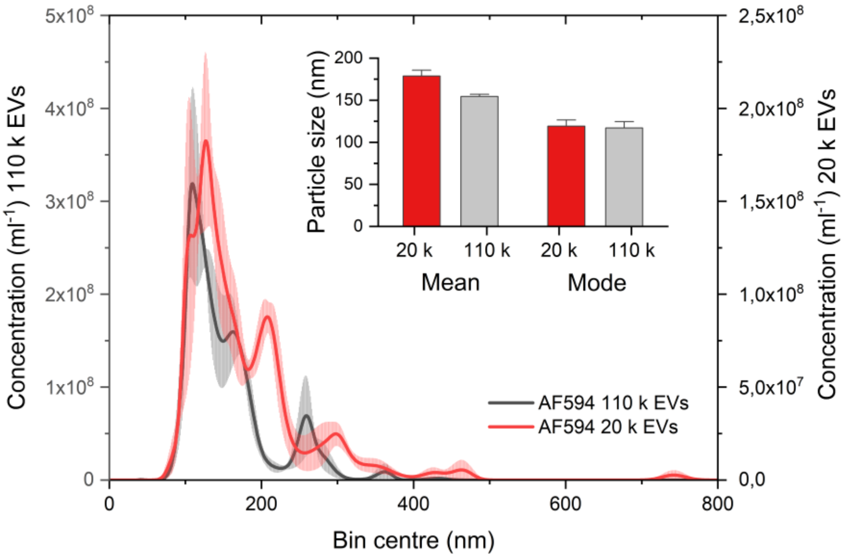

Figure S8. Representative NTA size distributions and particle average sizes (inset) for the AF594-labelled EVs after the SEC purification. The concentrations relate to 1:25 dilution of the original SEC fraction. The shaded area represents the standard error (N = 3).

Table S2. The characterization of the fluorescently labelled EVs after SEC in the highest EV concentration fraction (fraction 5): the particle concentrations determined by NTA, EV recovery, dye recovery, and labelling efficiency.

**Parameters in the SEC fraction 5**	**110 k EVs (*n* = 4)**	**20 k EVs (*n* = 4)**	**110 k EVs, inactivated AF594 (*n* = 1)**	**20 k EVs, inactivated AF594 (*n* = 1)**
EV concentration (ml^{-1})	$(2.1 \pm 0.5) \times 10^{10}$	$(1.4 \pm 0.3) \times 10^{10}$	2.3×10^{10}	1.9×10^{10}
EV recovery (%)	18.0 ± 4.2	12.1 ± 2.4	19.8	17.1
Dye recovery (%)	0.3 ± 0.1	0.3 ± 0.1	not detected	not detected
Labelling efficiency (molecules dye / EV)	481 ± 29	917 ± 169	-	-

S2. Polymeric nanoparticle dynamics in viscous solution

For comparison with the nanoparticle trajectories in the cells, we tracked FS100 in a simpler media, 1 % HEC, with 50 fps recording rate. Hydroxyethyl cellulose (HEC, Tylose H 10 000 P2, Shin Etsu, SE Tylose® GmbH & CoKG, Germany) in milli-Q water was mixed with FS100 diluted with milli-Q water to result in a final concentration of 1 % HEC and 10^9 particles ml^{-1}, and then carefully pipetted in a 10 × 10 mm gene frame chamber (ThermoFisher Scientific) mounted on a microscope slide. The chamber was sealed with a cover slip and equilibrated in room temperature before the tracking experiments.

HEC in aqueous solution forms a simple viscous solution, where the particles were expected to have purely diffusive movement. The ensemble-average MSD had anomality coefficient $\alpha = 1$ (**Figure S9b**) when fitted to Equation 6 (in the main article), and the shape of the PDF followed the definition of the normal diffusive displacements (**Figure S9c**), showing that the trajectories indeed were normally diffusive. The viscosity derived from the ensemble-average MSD (**Figure S9b**) and the Stokes-Einstein relation $D = k_B T (3\pi\eta d)^{-1}$ was 43 mPa·s. The analysis of the same image sequence by using TrackMate and TrajClassifier (parameters presented in **Table S3**) shows also almost only normal diffusion (95 % of the classified positions) with the diffusion constant close to identical to the result obtained with our Matlab script, both independent of the chosen analysis window size. Similar results between the two analysis approaches indicate that no segmenting is required for resolving the dynamics in this simple system. We compared the results to a macrorheological characterization of another 1 % HEC sample (**Figure S10**), which yielded a viscosity of 30 mPa·s. Since different samples were used in macro- and microrheological experiments, the difference between the results is likely related to small differences in concentration, and these two values were concluded to be in good agreement with each other.

Table S3. Experimental and analytical parameters for the segmented trajectory analysis of FS100 in 1 % HEC. One video recorded with 50 fps rate was analyzed with two analysis window sizes.

	**FS100 in 1 % HEC**	
Framerate (fps)	50	50
Video length (s)	10	10
Min. traj. length (s)	1	5
Analysis window (s)	1	5
Min. segment (s)	0.6	3
Tracks analyzed	1208	152

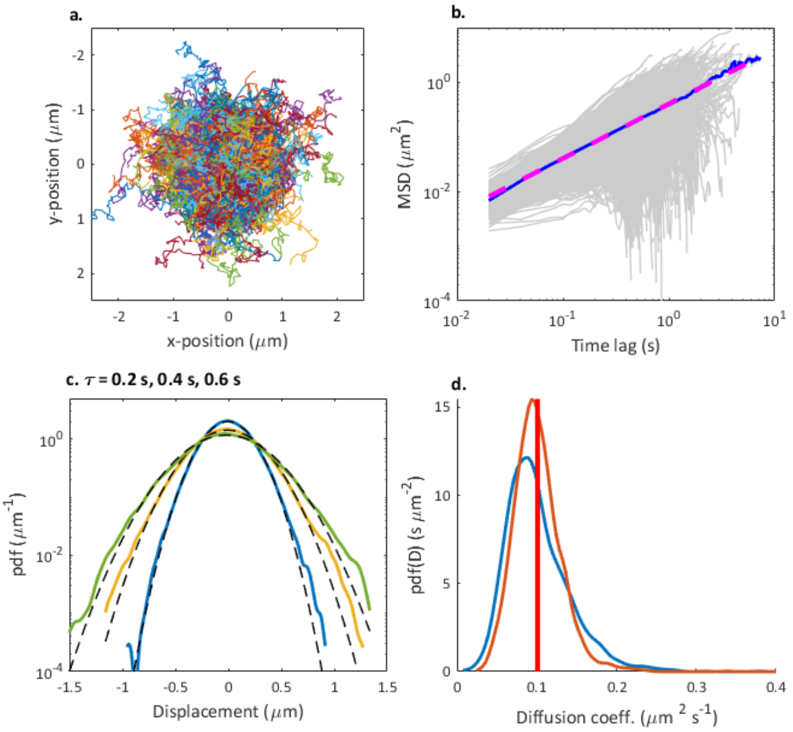

Figure S9. The summary of the analysis of FS100 in 1 % HEC, recorded with 50 fps. (a) Trajectories visualized with starting point at the origin. (b) Mean squared displacements. Grey curves are related to individual particle trajectories, blue is the ensemble average MSD, and magenta fit to the ensemble average. (c) Probability density function of particle displacements for three time lags (solid lines) and fit to the Gaussian distribution (dashed lines). (d) Distribution of diffusion coefficients. The analysis was repeated with the same number and similar length trajectories with simulated Brownian movement (shown in red). The red vertical line denotes the ensemble average and is overlapped with both experimental and simulated results. Figures (a–d) show that the FS100 behavior in 1 % HEC was Brownian, and the movement mode was recognized with our experimental setup.

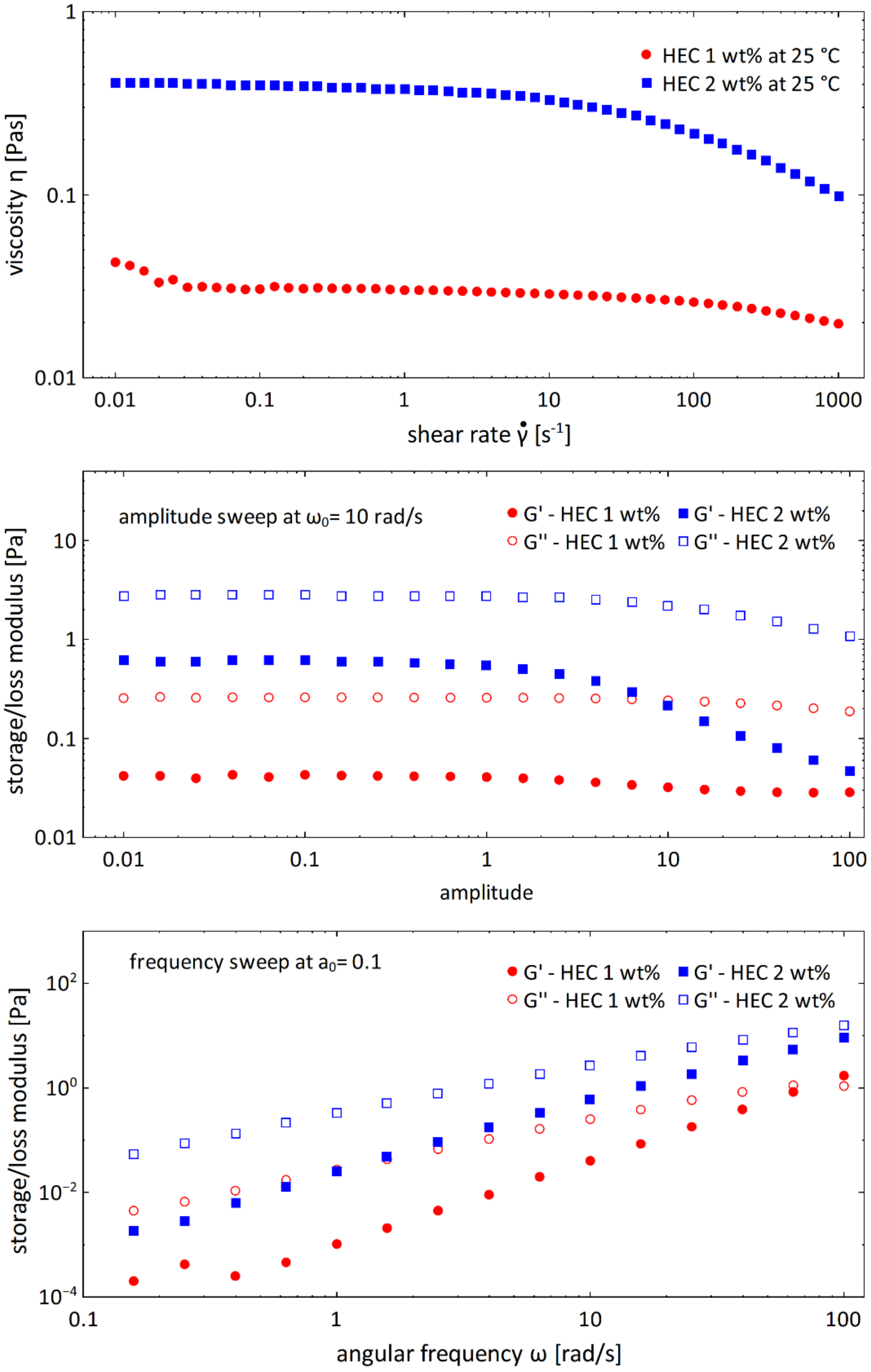

Figure S10. Macrorheological characterization of 1 % HEC and, for comparison, 2 % HEC in milli-Q water. The viscosity plot indicates almost no shear thinning. At the shear rate G’ of 1 s^{-1} the viscosity is 30 mPa·s for 1 % HEC and 375 mPa·s for 2 % HEC. The apparent G’ > 0 indicates a weakly elastic behaviour. However, the viscous behaviour is dominant as G’’ > G’. Experiments were done at 25 °C. Measurements by S. M. Recktenwald, Saarland University.

References

[1] K. Rautaniemi *et al.*, “Addressing challenges in the removal of unbound dye from passively labelled extracellular vesicles,” *Nanoscale Advances*, vol. 4, no. 1, pp. 226–240, Dec. 2022, doi: 10.1039/d1na00755f.
